# Does Subtelomeric Position of COMMD5 Influence Cancer Progression?

**DOI:** 10.3389/fonc.2021.642130

**Published:** 2021-03-09

**Authors:** Carole G. Campion, Thomas Verissimo, Suzanne Cossette, Johanne Tremblay

**Affiliations:** ^1^Centre de Recherche, Centre Hospitalier de l’Université de Montréal (CRCHUM), Montréal, QC, Canada; ^2^Département de Médecine, Université de Montréal, Montréal, QC, Canada

**Keywords:** COMMD proteins, COMMD5/HCaRG, kidney cancer, telomere, differentiation, biomarker, cellular senescence

## Abstract

The COMMD proteins are a family of ten pleiotropic factors which are widely conserved throughout evolution and are involved in the regulation of many cellular and physiological processes. COMMD proteins are mainly expressed in adult tissue and their downregulation has been correlated with tumor progression and poor prognosis in cancer. Among this family, COMMD5 emerged as a versatile modulator of tumor progression. Its expression can range from being downregulated to highly up regulated in a variety of cancer types. Accordingly, two opposing functions could be proposed for COMMD5 in cancer. Our studies supported a role for COMMD5 in the establishment and maintenance of the epithelial cell phenotype, suggesting a tumor suppressor function. However, genetic alterations leading to amplification of COMMD5 proteins have also been observed in various types of cancer, suggesting an oncogenic function. Interestingly, COMMD5 is the only member of this family that is located at the extreme end of chromosome 8, near its telomere. Here, we review some data concerning expression and role of COMMD5 and propose a novel rationale for the potential link between the subtelomeric position of COMMD5 on chromosome 8 and its contrasting functions in cancer.

## COMMD Proteins and Cancer

**Figure 1:**
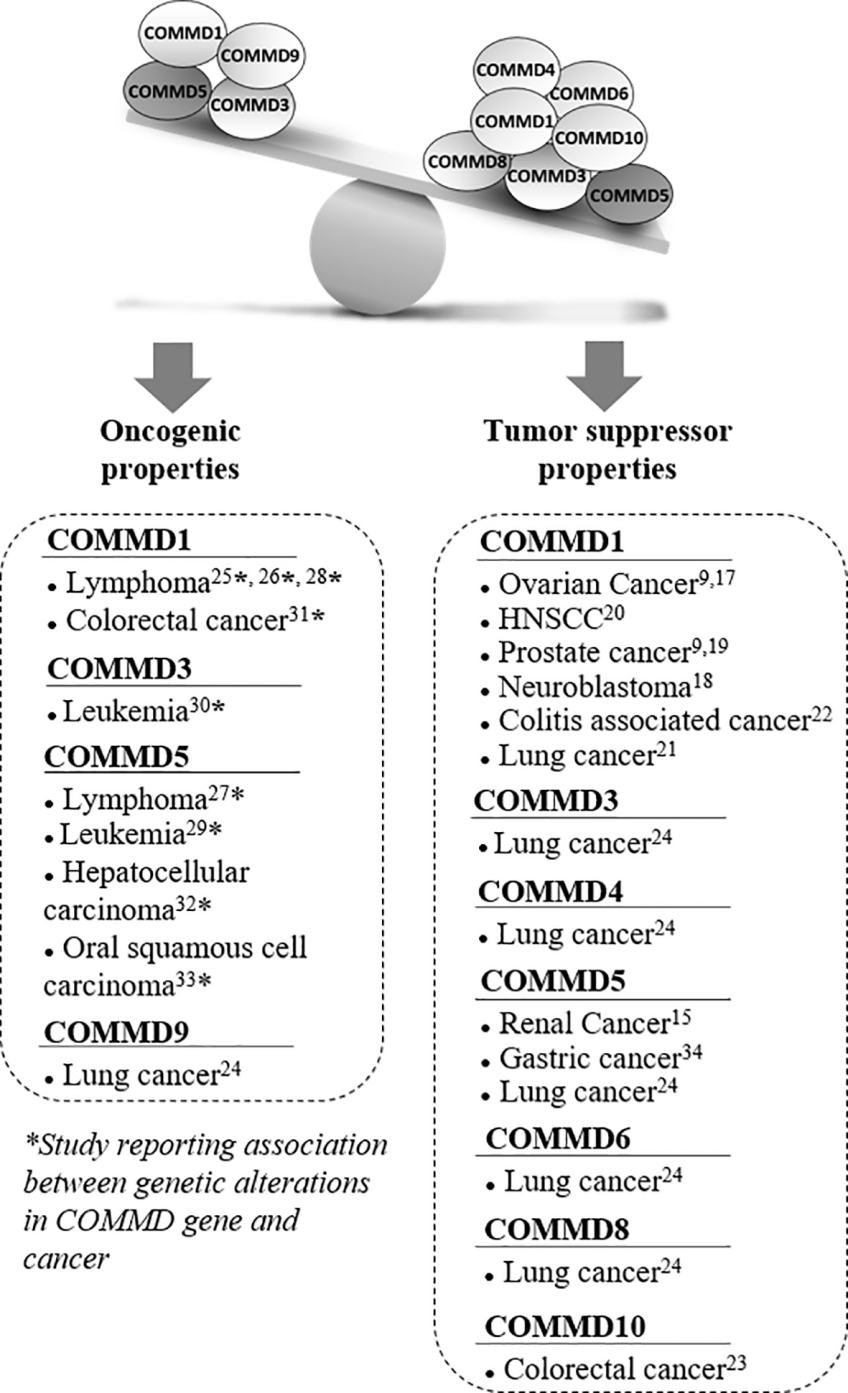
Associated studies to tumor suppressor or oncogenic properties of COMMD proteins. Several studies suggested that COMMD proteins had tumor suppressor properties, especially COMMD1 and COMMD5 that have been the most studied in cancer. Most of these studies reported lower *COMMD* expression levels in a variety of cancers that correlated with tumor progression. However, a few other publications reported some oncogenic properties and their potential involvement in tumor progression was always associated with chromosomal abnormalities and genetic alterations. Recurrent DNA amplifications and gain of chromosome material at COMMD1, COMMD5, and COMMD3 loci have been reported in various cancers. COMMD5 was the most frequently altered gene among the COMMD members to be associated to tumor progression.

COMMD proteins are part of a large multiprotein complex named Commander that contains up to 15 subunits including the CCC complex: COMMDs (1 to 10) proteins, CCDC22, and CCDC93, and three other components: C16orf62, SH3GLB1, and DSCR3 (1). This complex is highly conserved in vertebrates arguing that it is likely a complex of central importance involved in fundamental cellular function (1–3). COMMDs proteins have been reported in pleiotropic functions including, copper metabolism (4, 5), ubiquitination (6–8), hypoxia adaptation (9, 10), proinflammatory signaling (8, 11, 12), electrolyte transport (13), and endocytic sorting and recycling of various membrane proteins (4, 14–16). Only few studies have directly identified COMMDs proteins as therapeutic target in cancer and most of them reported a downregulation of COMMDs expression in cancer cells, suggested tumor suppressor properties (**Figure 1**). COMMD1 is the COMMD protein the most cited for its relation to cancer and decreased COMMD1 expression is associated with increased tumor invasion and worse survival (9). *COMMD1* expression was reduced in ovarian cancer (9, 17), neuroblastoma (18), prostate cancer (9, 19), head and neck squamous-cell carcinoma (HNSCC) (20), lung cancer (21), and colitis-associated cancer progression (22). As COMMD1 is a suppressor of both the NF-κB and HIF pathways which are transcriptional regulator of inflammation that plays an important role in oncogenesis, it is not surprising that COMMD1 expression has been correlated with patients’ survival in these different types of cancer (9). In colorectal cancer cells, COMMD10 also targets NF-κB (p65 subunit) and reduced its nuclear translocation, thereby leading to the inactivation of NF-κB pathway and cancer cells invasion and metastasis (23). The mRNA expression levels of COMMD3, COMMD4, COMMD5, COMMD6, and COMMD8 were also significantly downregulated in non-small cell lung cancer (NSCLC) cell lines, whereas COMMD9 was up-regulated and promotes the development of NSCLC by interacting with the TFDP1/E2F1 through the COMM domain (24).

Even if most studies related that decreased *COMMDs* expression was frequently observed in a variety of cancers and correlated with tumor progression, some publications suggested oncogenic properties (**Figure 1**). Interestingly, their potential involvement in tumor progression was associated to chromosomal abnormalities and genetic alterations. Studies have investigated the genetic basis of variations in gene expression associated with cancer susceptibility by performing whole genome array, single-nucleotide polymorphism array, and next-generation sequencing analyses. Molecular events were identified and associated with increased risk of malignancies, tumor relapse, and poor survival. They identified recurrent DNA amplifications, and gain of chromosomic region mapping the locus of COMMD1, COMMD5, and COMMD3 have been reported in lymphoma (25–28), leukemia (29, 30), colorectal cancer (31), hepatocellular carcinoma (32), and oral squamous cell carcinoma (33).

COMMD5/HCaRG is the second COMMD protein most published in relation to cancer. A down-regulation of COMMD5 has been observed in renal and lung cancer (15, 24) and in human gastric cancerous tissue (34). However, we noted that COMMD5 is also the most frequently altered gene among the COMMD member that was associated to tumor progression (27, 29, 32, 33). Surprisingly, these studies observed an amplification of COMMD5 that may paradoxically promote cancer progression.

## The Variable Expression of COMMD5 in Cancer

Twenty years ago, we identified a novel hypertension-related, calcium-regulated gene, HCaRG, that is overexpressed in different organs of genetically hypertensive strains of rats and whose expression is regulated by extracellular calcium levels with implications in cell proliferation. We mapped its gene on the distal end of human chromosome 8 (35–38). HCaRG was later shown to be COMMD5, the longest protein member of the COMMD family. Our studies demonstrated a role for COMMD5 in the establishment and maintenance of the epithelial cell phenotype, and suggested a tumor suppressor gene function (15, 35, 37–41). COMMD5 levels are low in various cancer cell lines in rodents and humans (35–38). We found that COMMD5 was underexpressed in human clear-cell renal cell carcinomas (CCRCCc) from 117 patients (39). Its expression was maintained in normal tissues adjacent to small renal tumors, while low expression was observed in normal adjacent tissues of larger size RCCs in patients with poor prognosis. Low COMMD5 levels in normal tissues were associated with worse clinical outcome (recurrence-free survival curves/5 years of patients) (39). COMMD5/HCaRG overexpression inhibited tumor growth and angiogenesis in a homograft renal carcinoma mouse model by promoting de-phosphorylation of ErbB2/HER2, ErbB3/HER3, and EGFR, leading to inhibition of ErbB signaling pathways (39). This suggests that add COMMD5 in a cell which loss its protein may reverse the differentiation state of cell which can return to a more differentiation state. Thus, COMMD5 expression is essential to maintain a differentiated state and events that induce downregulation of COMMD5 may lead to mesenchymal state of the cell.

**Figure 2:**
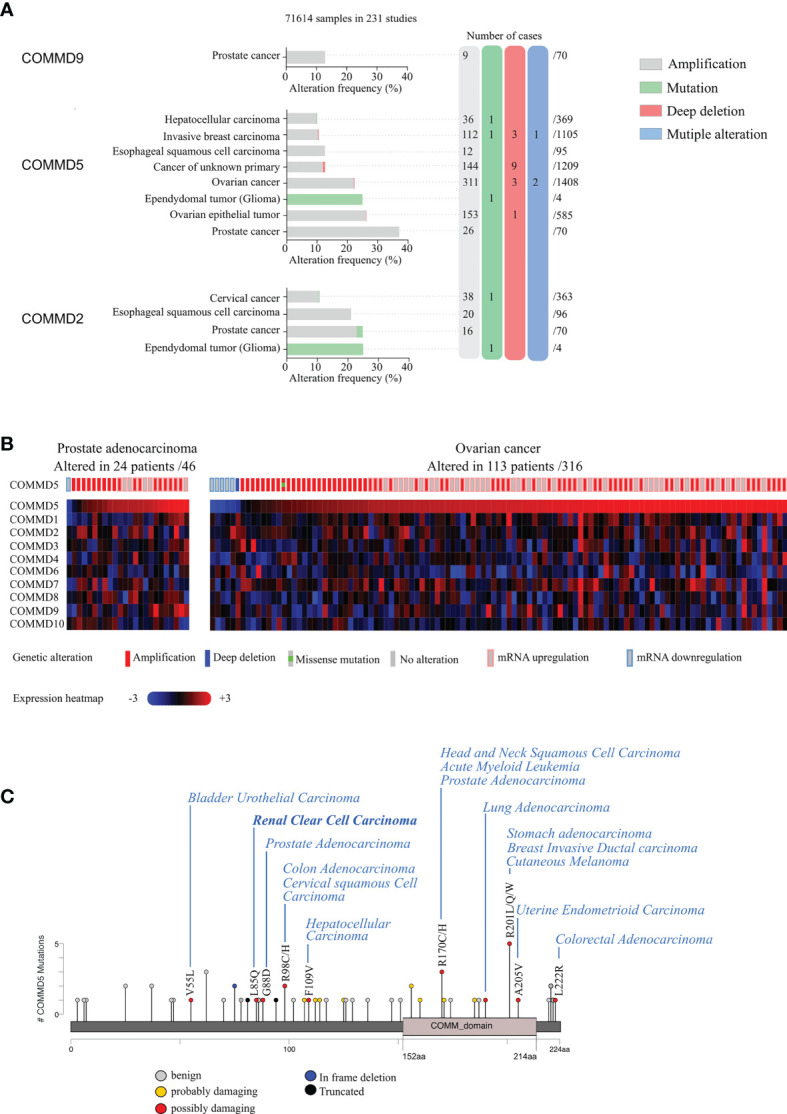
Genetic alterations and tissue expression profile of COMMD proteins and their association to different types of carcinoma. **(A)** *cBioportal database* was used to analyze mutations and copy number alterations of COMMD proteins in 231 studies (71,641 samples). Only COMMD2, COMMD5, and COMMD9 presented >10% CNV or mutations and were described in this figure. Genetic alterations of COMMD5 in different types of cancer showed the highest frequency of alterations in prostate and ovarian cancers. **(B)** Heatmap and copy number variations of COMMD proteins in ovarian cancer and prostate adenocarcinoma TCGA data (n = 46 and n = 316, respectively) obtained from cBioPortal. **(C)** Graphical summary of COMMD5 mutations from TCGA carcinoma studies mapped across the gene.

Interestingly, we also found that COMMD5 chromosomic alteration leading to COMDD5 amplification and overexpression was also associated to cancer progression (**Figure 2**). To deepen this new paradigm, we analyzed the type and frequency of mutations and copy number alterations (CNV) in the COMMD protein family reported in the cBioPortal database (42, 43). This analysis included data from 71,614 samples of different tumor types used in 231 studies. Interestingly, only COMMD2, COMMD5, and COMMD9 presented high rate of genetic alterations (>10% CNV or mutations) (**Figure 2A**). COMMD5 alterations have been detected in eight cancer studies, compared to four for COMMD2 and only one for COMMD9. The majority of COMMD5 genetic variations corresponded to amplifications and a very low frequency of gene mutation and deep deletions. High-level amplification of *COMMD5* was observed in prostate and ovarian cancers. To determine the impact of *COMMD5* amplification on its expression, we selected cancer studies with mRNA expression profiles. We found that copy-number gain of *COMMD5* strongly correlated with its mRNA upregulation in prostate and ovarian cancers (**Figure 2B**). None of the other COMMD members showed high mRNA level in these two types of cancers.

We also screened COMMD5 mutations in the 231 studies used above and evaluated their consequence on COMMD5 functions. Among the 44 reported mutations associated to cancer, 34 were located on sequences specific to COMMD5, and only 10 were within the COMM domain, a highly conserved 70–85 residue C-terminal domain shared by all COMMD members (**Figure 2C**). We used PolyPhen-2 (Polymorphism Phenotyping v2) tool and found that ~14% of mutations in COMMD5 specific region showed a high probability of damaging (high confidence) and ~9%, a potential probability of damaging (lower confidence) COMMD5 function. Among cancer associated to COMMD5 mutations were prostate, breast, lung carcinoma, leukemia, and RCC. We previously showed that COMMD5 is associated to differentiated cell phenotype and is downregulated in different cancer cell lines and RCC (38, 39). We therefore hypothesized that these COMMD5 mutations could disrupt COMMD5 gene or damaged COMMD5 function, leading to malignant cell conversion. Its location on chromosome 8q24, and its crucial role in cellular function makes COMMD5 a putative useful marker of kidney cancer progression and prognosis. Screening for COMMD5 expression levels and somatic mutations in cancer should be initiated.

Thus, these data suggest that both COMMD5 downregulation and upregulation may lead to cancer progression, leading us to propose some hypothesis that may explain this paradox.

## The Unique COMMD5 Subtelomeric Position

**Figure 3:**
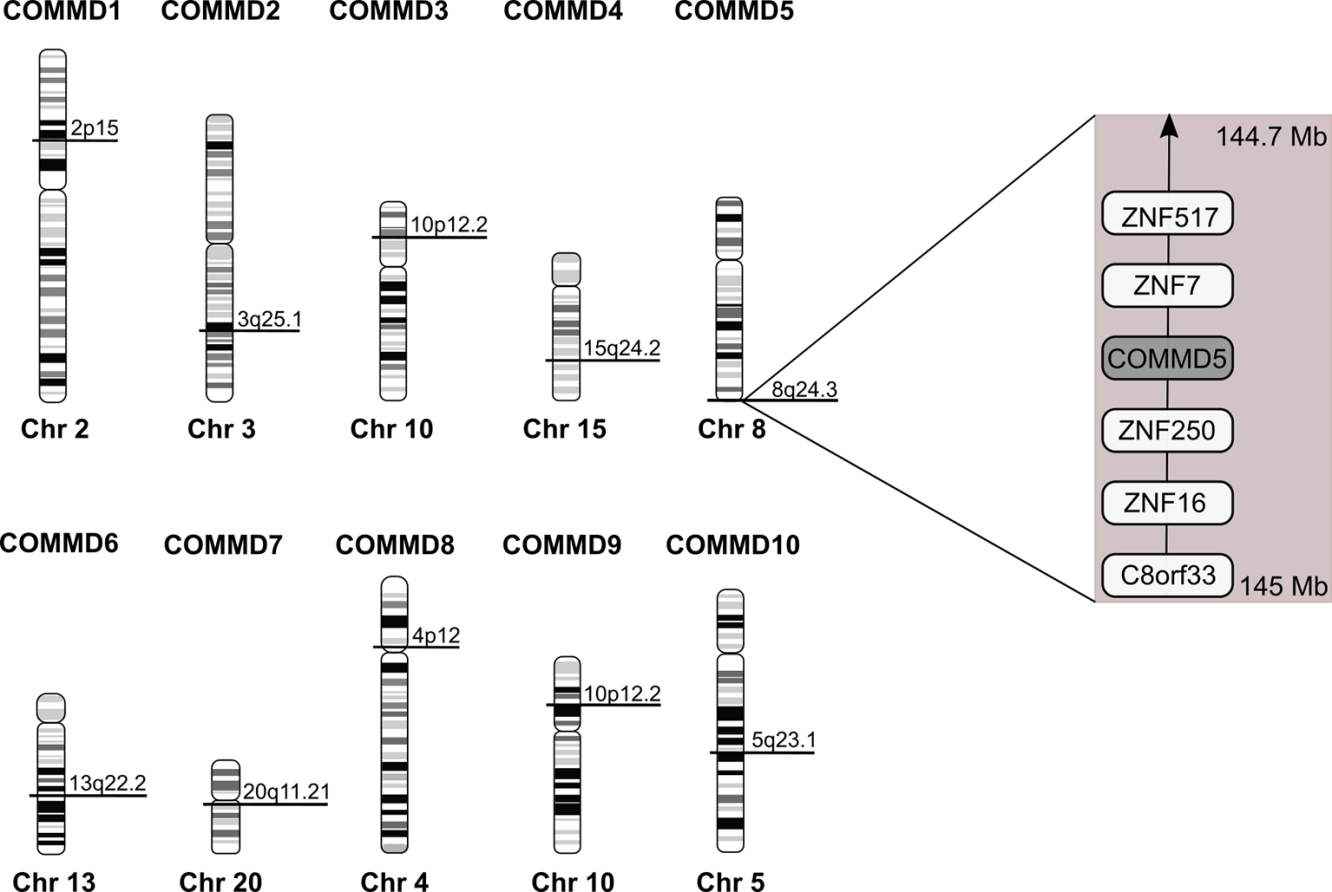
Chromosomal location of the human *COMMD* family members. Location of *COMMD* genes was deduced from the homo sapiens genome view of the *National Center for Biotechnology Information* (Gene ID: 150684 for *COMMD1*; 51122 for *COMMD2*; 23412 for *COMMD3*; 54939 for *COMMD4*; 28991 for *COMMD5*; 170622 for *COMMD6*; 149951 for *COMMD7*; 54951 for *COMMD8*; 29099 for *COMMD9* and 51397 for *COMMD10*). COMMD5 is the only COMMD family member located at the extreme end of chromosome. According to *The Human Protein Atlas database*, COMMD5 is the fourth protein-coding gene from the end of 8q24.3 chromosome.

Among the COMMD family, COMMD5 is the only one located at the extreme end of chromosome 8, 8q24.3 (**Figure 3**). COMMD5 is the fourth coding protein before the end of the chromosome. In order to explain the variable levels of expression of COMMD5 in cancer, we assessed whether its localization at the end of chromosome 8 could regulate its level of expression in different cancers and during ageing process.

## Chromosome 8q24.3 Alterations and Cancer Susceptibility

Genome wide association studies (GWAS) have identified a large number of single nucleotide polymorphisms (SNPs) and the majority of common risk alleles discovered to date map outside of known protein coding regions (e.g., intronic and intergenic regions). However, a particularly interesting set of risk loci is clustered within the 8q24 region (chr.8q24) and is linked to susceptibility to different cancers including kidney (44), prostate (45–47), breast (48–50), gastric (51), colon (52–54), ovarian (55, 56), bladder (57), and chronic lymphocytic leukemia (58). In addition, the 8q24 region has recently been identified in a large-scale study across human cancers as the most frequently amplified region (59). One gene found within this chr.8q24 region, MYC, is the most frequently amplified protein-coding gene across all cancer types (59).

Few genetic studies have related copy number variants (CNV) and COMMD5 transcripts to cancer progression. Using Affymetrix SNP 6.0 and Affymetrix GeneChip Human Gene 1.0 ST arrays, Peng et al. have identified recurrent DNA amplifications scattered from 8q22.2 to 8q24.3 in 112 Oral Squamous Cell Carcinoma (OSCC) specimens (33). COMMD5 was a gene within these amplicons that might be critical to OSCC progression and these DNA amplifications significantly associated with poor survival, and possible early development of second primary tumors. In an integrative genomic analysis of a large series of patients with fibrolamellar hepatocellular carcinoma (FLC) using next-generation sequencing, SNP-array and whole-transcriptome analysis, the most frequent focally amplified locus was at 8q24.3 in 4/32 patients (12.5%) spanning several genes including *COMMD5* (32). High-resolution cytogenetic techniques that combine laser capture micro-dissection with microarray-based comparative genomic hybridization technology have provided new opportunities to investigate genome-wide DNA alterations in limited-sized lesions (60, 61). Using these technologies, Slovak et al. compared the Hodgkin lymphoma molecular karyotypes to the genomic profiles of germinal center B cells and treatment outcome (chemotherapy responsive vs. primary refractory disease) (27). Among the most frequent gains (>65%), they identified the 8q24.3 region which includes genes associated with growth and proliferation. Among them, COMMD5 was identified. Finally, a recent study examined copy number aberrations in the subtelomeric regions of a patient with *de novo* acute monocytic leukemia (29). This study reported that COMMD5 locus was in the thirty one out of 92 subtelomeric regions (33.7%) which had duplications between 141,682 and 864,400 bp in size.

**Figure 4:**
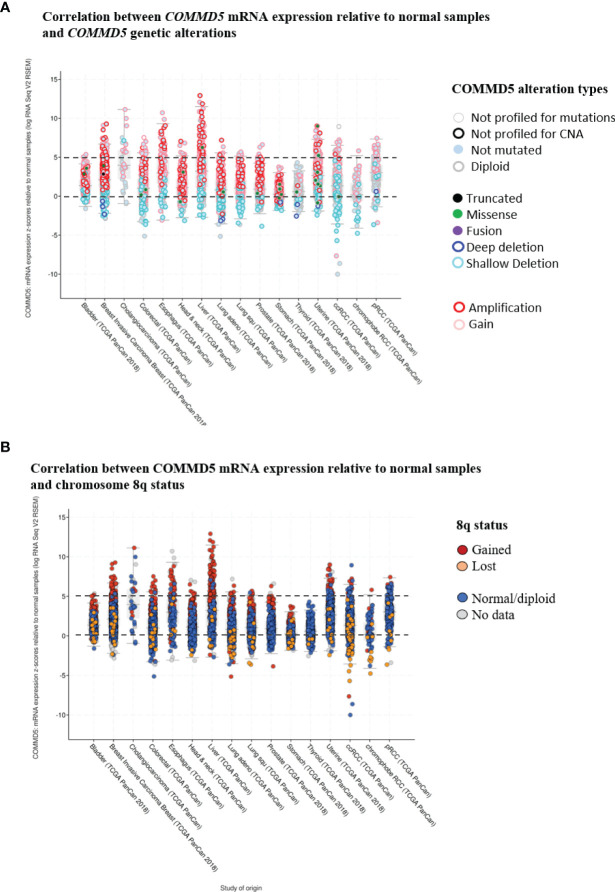
Correlation between *COMMD5* mRNA expression relative to normal samples and *COMMD5* genetic alterations or chromosome 8q status in several cancer types. cBioportal database (42, 43) was used to analyze COMMD5 mRNA expression relative to normal samples in several cancer types using data generated by the Cancer Genome Atlas (TCGA). This analysis included data from 10,967 samples of different human tumor types used in 32 studies. **(A)** Plot showing correlation between *COMMD5* mRNA levels relative to normal samples and *COMMD5* genetic alteration in several cancer types. As indicated in the cBioportal database: Deep deletion indicates a deep loss, possibly a homozygous deletion; Shallow deletion indicates a shallow loss, possibly a heterozygous deletion; Gain indicates a low-level gain (a few additional copies, often broad); Amplification indicates a high-level amplification (more copies, often focal). **(B)** Plot showing correlation between *COMMD5* mRNA expression relative to normal samples and chromosome 8q status in several cancer types (including 8q amplification “gained”, 8q deletion “lost”, no changes in 8q status “normal diploid” and no data). Abbreviations: Lung adeno, lung adenocarcinoma; Lung squ, lung squamous cell carcinoma; ccRCC, clear cell renal cell carcinoma; pRCC, papillary renal cell carcinoma.

Gain of 8q24 region is frequently observed in genome wide association studies (GWAS) of cancer. Through its position on chromosome 8q24.3, COMMD5 is clearly a target for copy-number alterations, and thus a candidate gene for cancer susceptibility. Indeed, using cbioportal database (42, 43), we analyzed *COMMD5* mRNA expression relative to normal samples (non-cancerous samples) in several cancer types using the data generated by the Cancer Genome Atlas (TCGA). This analysis included data from 10,967 samples of different human tumor types used in 32 studies. Firstly, we did a correlation analysis between *COMMD5* mRNA expression and *COMMD5* genetic alterations (**Figure 4A**). The plot analysis showed that occurrence of COMMD5 amplification is frequently observed in most of cancer types and particularly in breast invasive carcinoma, oesophagus, liver, uterine, and renal cancers, where this amplification correlated with high level of *COMMD5* mRNA expression (more than five-fold relative to normal samples). We also found that several shallow deletions and some deep deletions correlated with *COMMD5* mRNA downregulation and this is more pronounced in breast invasive carcinoma, colorectal, lung and renal cancer including ccRCC and chromophobe RCC (**Figure 4A**). We next investigated the relationship between *COMMD5* mRNA expression (over or under-expression) and chromosome 8q alterations including 8q amplification (“gained”) or 8q deletion (“lost”) in these tumors (**Figure 4B**). Interestingly, gain of chromosome 8q were noted in cancers with higher levels of *COMMD5* mRNA, including breast invasive carcinoma, oesophagus, liver, and uterine cancers. Furthermore, downregulation of *COMMD5* expression correlated with 8q loss in invasive breast cancer, colorectal and lung cancers and in ccRCC and chromophobe RCC.

Altogether these data support our hypothesis that *COMMD5* expression may be influenced by chromosome 8q alterations, thus, we next investigated whether its proximity to telomere could influence the up- as well as downregulation of *COMMD5* gene expression levels.

## Is COMMD5 Expression Controlled by Its Subtelomeric Position?

The extreme ends of eukaryotic chromosomes, the telomeres, are special structures that provide protection from enzymatic end-degradation and are crucial in the maintenance of chromosome integrity and genomic stability (62). During cell division throughout life, telomeres are progressively shortened and when telomeres reach a threshold length, a DNA damage response is triggered, leading cells to enter in senescence or in apoptosis (63, 64).

**Figure 5:**
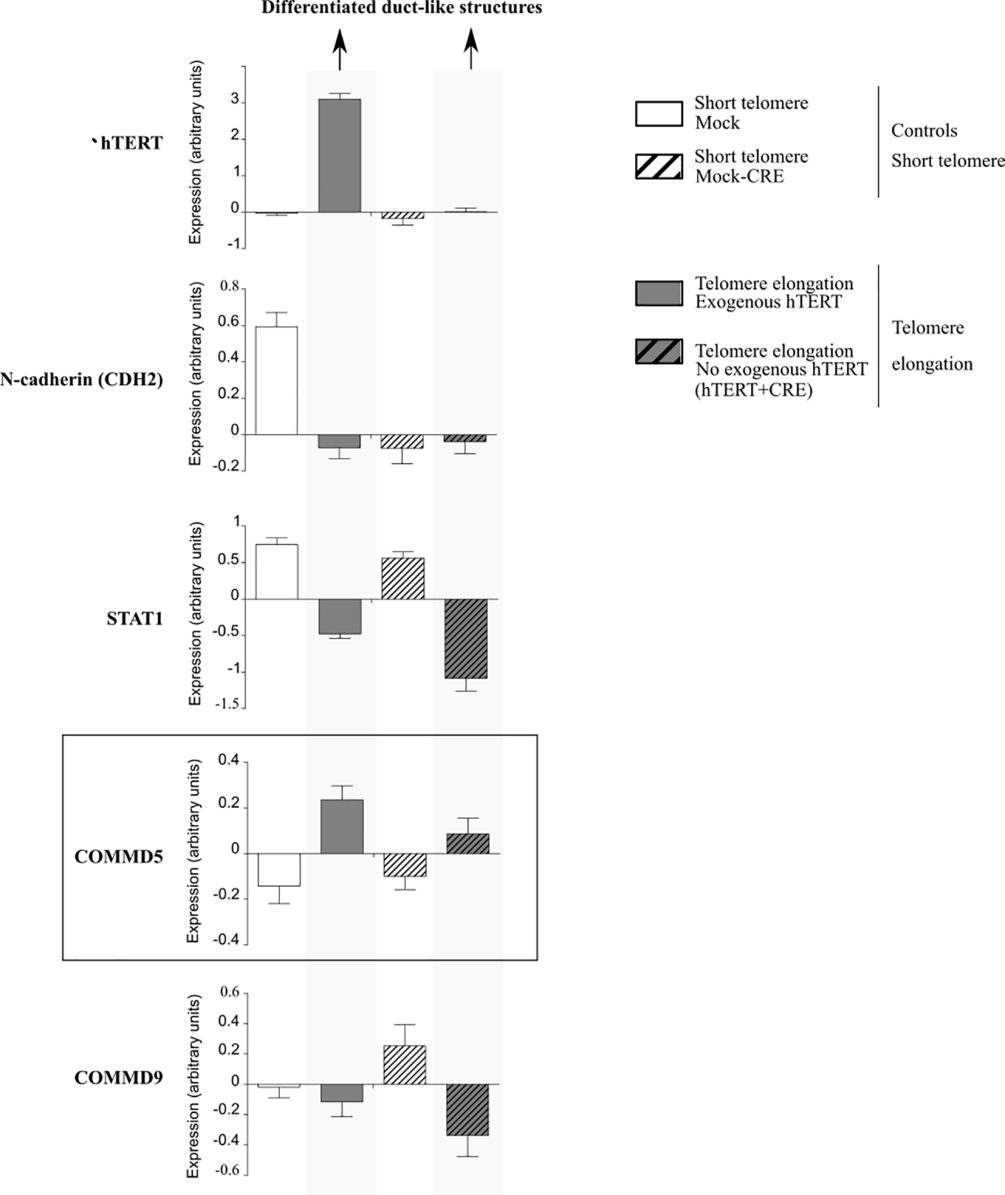
Analysis of mRNA expression profiles in PC-3 prostate cancer cells in the presence of exogenous human telomerase reverse transcriptase (hTERT). Forced elongation of telomeres correlated with *COMMD5* expression. Data were extracted from the geoprofile database (GSE41559), plotted in an excel file and analyzed. Hirashima et al. (62) established a PC-3 sub-line that overexpressed exogenous hTERT (hTERT) and upregulation of telomerase activity and substantial telomere elongation in these PC-3/hTERT cells was compared with control cells (Mock). To examine whether the formation of the duct-like structures resulted from telomere elongation and not from increased levels of hTERT protein, they removed the hTERT transgene after telomere elongation using the Cre/*loxP* system. They added the *loxP* sequence at both the 5′ and 3′ ends of the wild-type hTERT cDNA and established the stable PC-3/LhTERTL cell line (hTERT+CRE) or control cells (Mock-CRE). They subcutaneously injected these four PC-3 cell lines, mock, hTERT, mock-CRE, and hTERT+CRE, into nude mice and collected the resultant xenograft tumors to monitor gene expression that might be important for differentiation of PC-3 cells *in vivo* using a microarray approach. Forced elongation of telomeres in cancer cells promotes PC-3 cell differentiation and the mRNA expression of *N-cadherin*, *STAT1* and *COMMD5* but not *COMMD9*.

Firstly, it is tempting to propose that COMMD5 functions could be related to the subtelomeric position of its gene as studies suggested that telomere length influences cell differentiation (62, 65). In this context, our previous studies demonstrated that COMMD5 plays an essential role in the establishment and maintenance of the epithelial cell phenotype (35–38). Furthermore, we showed that COMMD5 overexpression in kidneys accelerated tubular repair after ischemic injury of transgenic mice by modulating renal cell proliferation and migration, and by facilitating their re-differentiation (40, 41). This is in line with Westhoff et al. who demonstrated that short telomeres are associated with an increased renal injury and decreased recovery (66). Hirashima et al. (62) used PC-3 (prostate cancer) cells exhibiting short telomeres and forced their elongation by enhancing cellular telomerase activity. They observed that telomere elongation in these cells resulted in the formation of duct-like structures and well-differentiated tumors *in vivo*. We analyzed *COMMD5* mRNA expression in this study by using data from Gene expression omnibus (GEO) profile GSE41559 (**Figure 5**). In most cases, *COMMD5* expression inversely correlated with the expression of *N-cadherin*, a mesenchymal marker and with *STAT1*, an immune response-related gene in the tumor microenvironment while *COMMD9* expression, whose chromosomic location is not in telomeres did not correlate with cancer cell differentiation status. This novel observation showed that high levels of *COMMD5* correlated with 1) telomere elongation (by hTERT overexpression and by hTERT+CRE), and 2) cell differentiation induced by telomere elongation. Pucci et al. (65) also showed that functional telomeres are important for the stability of stem cell differentiation as short telomeres in embryonic stem cells led to unstable differentiation. Cancer cells maintain shorter telomeres than the cells in the surrounding normal tissues to sustain their undifferentiated state. We have shown that higher COMMD5 protein levels in normal tissue surrounding RCC tumors favored their differentiated phenotype, reduced tumor growth and enlargement, and correlated with survival rate and better prognosis of patients with RCC (39). Interestingly, many studies observed shorter telomere length in RCC tumors compared with paired normal tissue (67–72). Pal et al. (73) analyzed 100 cases of RCC for telomerase activity and found that RCC tissues had significantly shorter telomere length than the adjacent normal parenchyma. They also found a correlation between telomere length and grades (p = 0,016) of ccRCC but not with its stages (0,20) or subtypes (p = 0,67): low-grade tumors had significantly longer telomeres than high grades which correlated with reduced telomere length. Furthermore, shortening of telomeres has been shown to contribute to renal abnormalities including, glomerular senescence, impaired potassium clearance, renal cysts, fibrosis, glomerulosclerosis, and renal cell carcinomas (74).

Secondly, although telomere shortening can lead to genetic instability and is often correlated with the onset of diseases and cancers, many studies have provided evidence that long telomeres can also contribute to cancer development (75, 76). Indeed, even if telomere attrition imposes a barrier to cell proliferation, some cancers developed an adaptative response and can bypass DNA damage response pathways and cellular senescence by upregulating telomerase, a cellular ribonucleoprotein enzyme complex whose function is to elongate telomeres (77). Thus, the association between telomere length and the risk of cancer remains conflicting and these observations suggest that telomeres may play diverse roles in different type of cancers. In renal cancer, Morais et al. proposed that telomeres may play a dual role during RCC carcinogenesis; in the early stages, short telomeres may increase RCC risk and in late carcinogenesis, long telomeres seem to be associated with bad tumor prognosis (71). Using a large series of colorectal cancers, Rampazzo et al. demonstrated that telomere length varies not only with tumor stage but also differs according to tumor location, being longer in rectal cancers (*p* = 0.03) (78). They also demonstrated that telomeres were significantly shorter in colorectal than in adjacent non-cancerous tissues, regardless of tumor stage, grade, site, or genetic alterations. Hence, they proposed that the different telomere lengths in cancers may be due to different kinetics of telomere erosion/stabilization.

Considering these observations, we propose that the variability of telomere length in different type of cancers could explain the variable expression of COMMD5 in cancers. This hypothesis is strengthened by the concept of telomere position effects over long distances, TPE-OLD, a mechanism by which gene expression is modulated by telomere length dependent loops (79–82). These telomere loop structures bring genes in direct proximity to the telomeres and can extend to at least 10 Mb from the chromosome end. Studies demonstrated that TPE-OLD induces a local modification of chromatin organization leading to transcriptional changes of genes in close proximity to the loop (80, 83, 84). This phenomenon is explained by the fact that TPE-OLD involve chromatin modifications (acetylation, methylation) and chromatin remodeling factors that influence gene in direct proximity to this telomere loop (85–89). Upon telomere shortening, looping diminishes, separating the TPE-OLD genes from the telomere and its chromatin signature, inducing a new transcriptional modulation of neighboring genes (79). Loop disruption occurs long before telomere shortening induces DNA damage responses.

Thus, TPE-OLD is an active mechanism that participates in the regulation of gene expression by upregulating or down-regulating their expression. We suggest that TPE-OLD could be one of the mechanisms responsible for differential transcriptional levels of COMMD5 in cancer.

## Could COMMD5 Subtelomeric Position Influence Cell Senescence?

**Figure 6:**
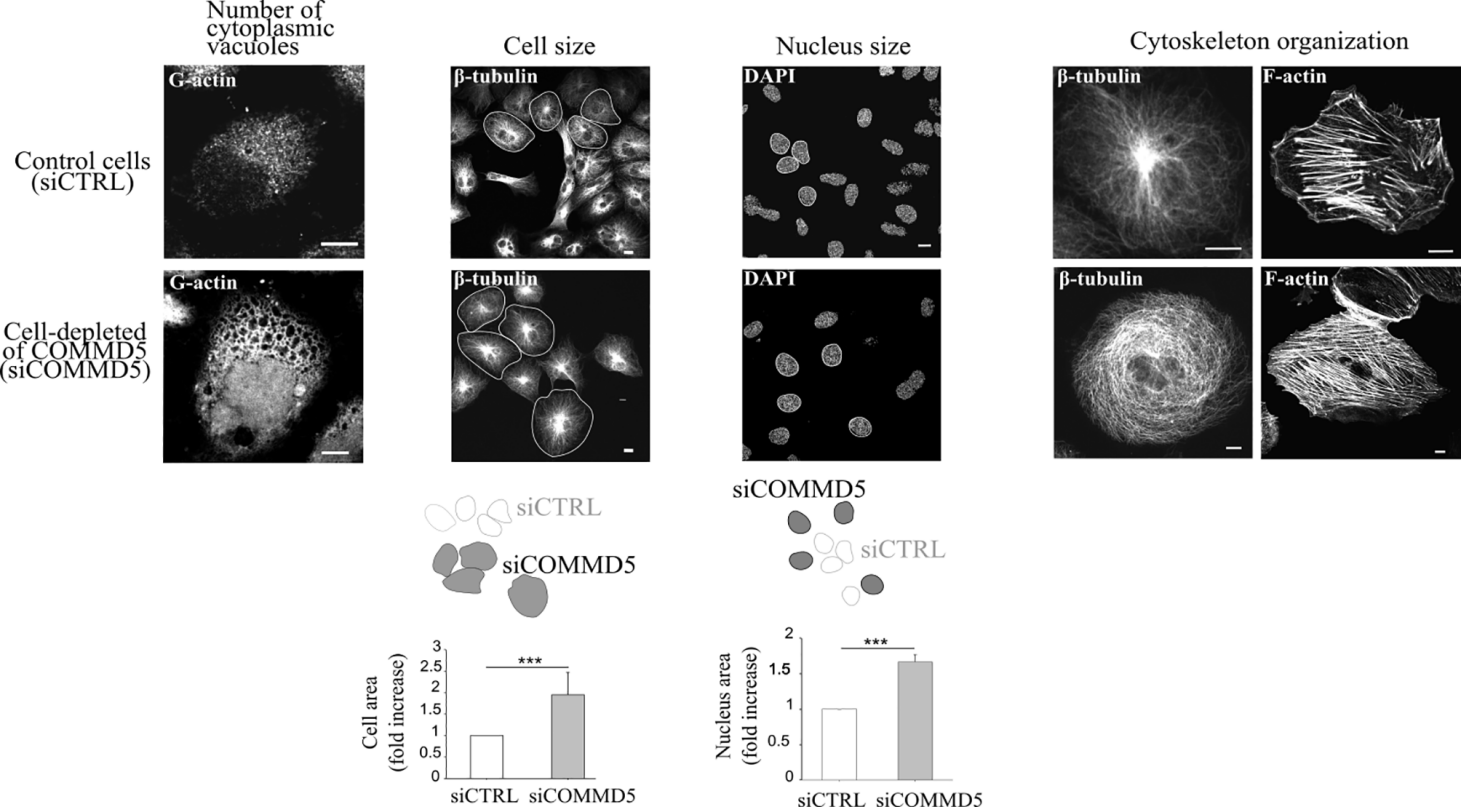
Loss of COMMD5 leads to cellular morphological changes including cytoskeleton organization. HK-2 cells were transfected with COMMD5 specific siRNA (siCOMMD5) or control siRNA (siCTRL), fixed, and labeled with fluorescein-deoxyribonuclease 1 (for G-actin), β-tubulin, phalloidin (for F-actin) or DAPI (for nuclei staining). Cell size or nucleus area in cells transfected with siCTRL or siCOMMD5 were processed and analyzed using FIJI software (values are means, error bars indicate SD, n = 3). Student’s t test, ***p < 0.001, significant differences compared with siCTRL.

It has been proposed that when telomeres shorten to a critical point, a signal is sent to stop further cell division, the hallmark of cellular senescence. The subtelomeric position of COMMD5 raises the question whether loss of COMMD5 after telomere shortening could participate to cellular senescence. Cellular senescence refers to the irreversible arrest of cell proliferation (growth) (64, 90). We reported previously (15) that proliferation of renal cell lines (HK-2) depleted of COMMD5 by specific siRNA, is stopped without DNA fragmentation or cell mortality. Morphological change is one of the featured characteristics of senescence. Morphological changes that accompany replicative senescence are increased in cell, nuclear, and nucleolar size, presence of multinucleated cells, prominent Golgi apparati, higher number of vacuoles in the endoplasmic reticulum and cytoplasm, more cytoplasmic microfilaments, and large lysosomal bodies (91, 92). Size of senescent cells could be twice as much as non-senescent ones (93). We found that COMMD5 loss induced important morphological changes including higher number of cytoplasmic vacuoles, a rounded cell shape with a doubled cell size and 1.5 fold larger cell nucleus [**Figure 6** and (15)]. In addition, COMMD5 depletion led to a strong cytoskeletal re-organization with an enrichment of actin stress fibers and a disorganized distribution of microtubules that lose their orientation and acquire an equal radial distribution [**Figure 6** and (15)]. These characteristics have also been reported by Xu et al. (94) who showed that miR-22 repressed cancer progression by inducing cellular senescence. They found a significant difference in cell size (up to 1.6-fold) between senescent cells induced by miR-22–treatment and control cells. In addition, miR-22–treated cells contained larger actin stress fibers and the authors proposed that miR-22–induced senescence morphology in cancer cells reduced cell motility and invasion. They also observed that senescent fibroblasts and Lenti-Pre22–infected cancer cells exhibited large flattened senescence-like morphology that reduced cell movement. We have also observed these features after COMMD5 depletion in human kidney (HK-2) cell lines. COMMD5-depleted cells had flattened and enlarged cell shapes and exhibited a random migration with most of them spinning around themselves [Video S8 in supplemental information of (15)]. Cells depleted of COMMD5 lost their directional movement leading to a shorter distance of migration and reduced cell motility. As also observed by Xu et al. (94) in their study, the strong accumulation of actin stress fibers in the cortical region and the loss of microtubule orientation detected in COMMD5-depleted cells probably caused their rounded shape, thus abolishing their directional movement.

Senescence is a stress response that can be induced by a wide range of intrinsic and extrinsic insults, including oncogenic activation, oxidative stress, telomere shortening, etc. (63). In this later event, a DNA damage response is first necessary before cells enter into an early senescence phase. However, COMMD5 subtelomeric position combining to TPE-OLD mechanism could create a favourable environment for the downregulation of COMMD5 and induction of senescent-like features in cells long time before cells inducing a DNA damage response.

## Conclusion

**Figure 7:**
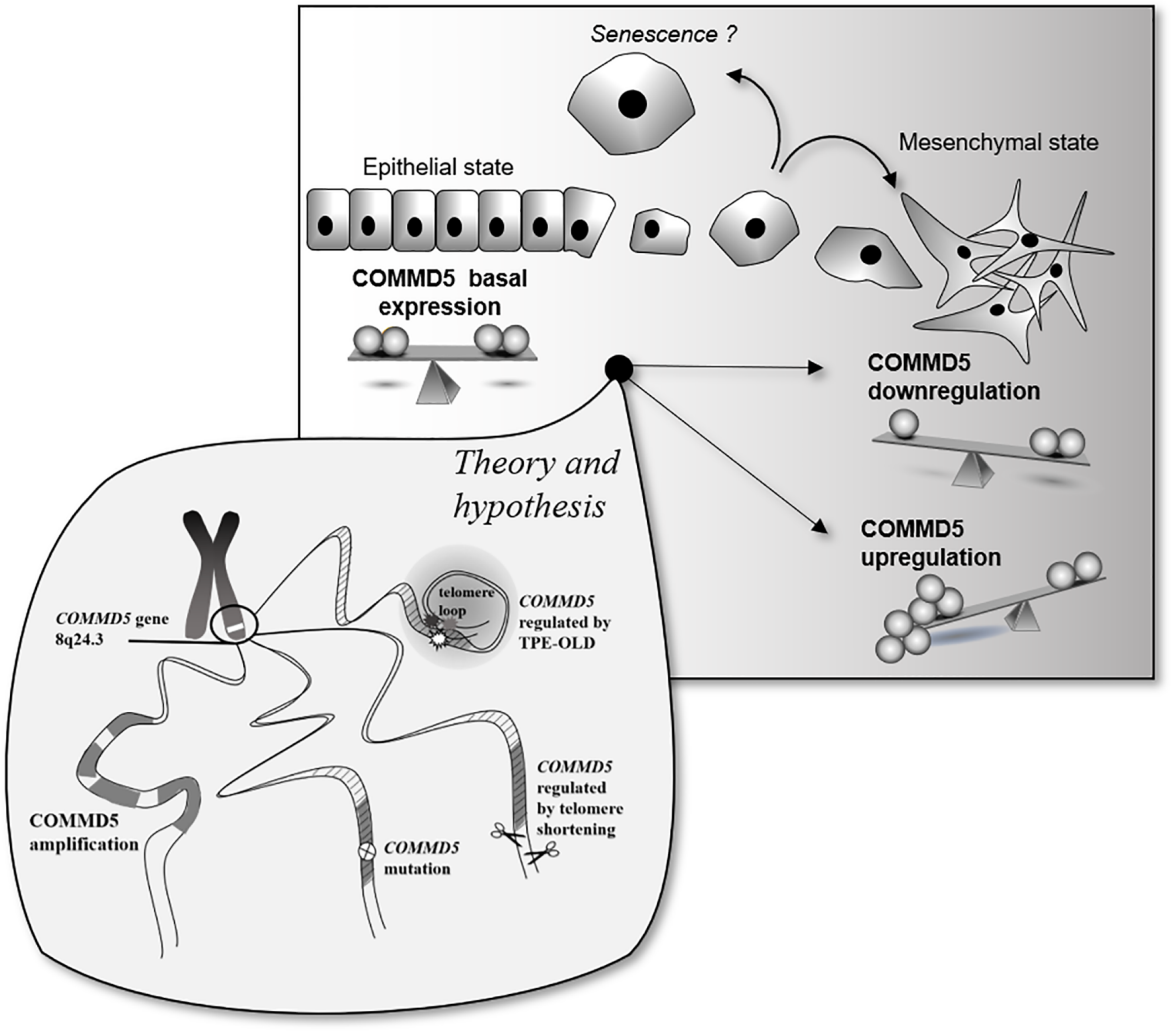
Schematic representation of putative COMMD5 expression profile and cell status. COMMD5/HCaRG plays an essential role in the establishment and maintenance of the epithelial cell phenotype. Downregulation of COMMD5 or complete loss of COMMD5 expression or function by mutations induce an epithelial to mesenchymal transition. On the other hand, too much of COMMD5 is also deleterious to cells. COMMD5 is localized at the extreme end of the chromosome 8, a region highly targeted by locus amplification. We propose here that its subtelomeric localization could explain the variable expression of COMMD5 in cancer. Gene amplification, mutation, and proximity to telomere are all events that may contribute to regulation of COMMD5 expression in cancer. Consequently, as COMMD5 is involved in crucial cellular functions such as cell differentiation, proliferation, and migration, expression of this gene must be tightly controlled.

The novel observation that COMMD5 expression could be differently regulated in cancer cells by its locus alterations, amplifications, mutations or by telomere length, raises several questions with regards to its function and association to cancer. Is COMMD5 overexpression or downregulation good or bad? Is COMMD5 an oncogenic factor or a tumor suppressor gene? Is COMMD5 a promising therapeutic target for cancer therapy? COMMD5 is expressed in all epithelial tissues and we previously found that its basal expression is essential to maintain a differentiated cell phenotype. The data presented here demonstrate the duality of COMMD5 expression from down to upregulation, but both correlating with cancer susceptibility. Molecular mechanisms that modulate COMMD5 expression in cancer have not yet been elucidated, offering a wide range of possibilities. Here, we focused on the localization of COMMD5 at the subtelomeric position of the chromosome and developed a rationale that this could impact on COMMD5 gene regulation in cancer cells. We showed that COMMD5 gene expression could be affected by different chromosomal events including gene amplification, mutation and telomere length. Upregulation or downregulation of COMMD5 expression by one of these events may elicit cells to undergo mesenchymal transition or even, to acquire senescence-like phenotype. Occurrence of these different events may vary according to tumor type, stage and location. In conclusion, as COMMD5 is involved in carcinogenesis probably by regulating cell differentiation and playing crucial roles in wound healing or tissue regeneration, its expression must be tightly regulated and controlled. So, small differences in COMMD5 expression could induce variations in cancer susceptibility, and its functional properties are strongly related to its level of expression (**Figure 7**).

## Data Availability Statement

Publicly available datasets were analyzed in this study. This data can be found here: https://www.cbioportal.org/.

## Author Contributions

CC conceived, designed, performed the figures and data analyses, and wrote the manuscript. JT, SC, and TV critically reviewed the manuscript and JT supervised its achievement. All authors contributed to the article and approved the submitted version.

## Funding

Our study was funded by the Canadian Institutes of Health Research (CIHR) (grant MOP-133690) to JT. CC was supported by a KRESCENT postdoctoral fellowship.

## Conflict of Interest

The authors declare that the research was conducted in the absence of any commercial or financial relationships that could be construed as a potential conflict of interest.

## References

[1] Mallam AL Marcotte EM. Systems-wide Studies Uncover Commander, a Multiprotein Complex Essential to Human Development. Cell Syst (2017) 4:483–94. 10.1016/j.cels.2017.04.006 PMC5541947 28544880

[2] Burkhead JL Morgan CT Shinde U Haddock G Lutsenko S. COMMD1 forms oligomeric complexes targeted to the endocytic membranes *via* specific interactions with phosphatidylinositol 4,5-bisphosphate. J Biol Chem (2009) 284:696–707. 10.1074/jbc.M804766200 18940794 PMC2610505

[3] Healy MD Hospenthal MK Hall RJ Chandra M Chilton M Tillu V. Structural insights into the architecture and membrane interactions of the conserved COMMD proteins. Elife (2018) 7:e35898. 10.7554/eLife.35898 30067224 PMC6089597

[4] Phillips-Krawczak CA Singla A Starokadomskyy P Deng Z Osborne DG Li H. COMMD1 is linked to the WASH complex and regulates endosomal trafficking of the copper transporter ATP7A. Mol Biol Cell (2015) 26:91–103. 10.1091/mbc.e14-06-1073 25355947 PMC4279232

[5] van De Sluis B Rothuizen J Pearson PL van Oost BA Wijmenga C. Identification of a new copper metabolism gene by positional cloning in a purebred dog population. Hum Mol Genet (2002) 11:165–73. 10.1093/hmg/11.2.165 11809725

[6] Drevillon L Tanguy G Hinzpeter A Arous N de Becdelievre A Aissat A. COMMD1-mediated ubiquitination regulates CFTR trafficking. PLoS One (2011) 6:e18334. 10.1371/journal.pone.0018334 21483833 PMC3069076

[7] Ganesh L Burstein E Guha-Niyogi A Louder MK Mascola JR Klomp LW. The gene product Murr1 restricts HIV-1 replication in resting CD4+ lymphocytes. Nature (2003) 426:853–7. 10.1038/nature02171 14685242

[8] Maine GN Burstein E. COMMD proteins: COMMing to the scene. Cell Mol Life Sci (2007) 64:1997–2005. 10.1007/s00018-007-7078-y 17497243 PMC2938186

[9] van de Sluis B Mao X Zhai Y Groot AJ Vermeulen JF van der Wall E. COMMD1 disrupts HIF-1alpha/beta dimerization and inhibits human tumor cell invasion. J Clin Invest (2010) 120:2119–30. 10.1172/JCI40583 PMC2877941 20458141

[10] van de Sluis B Muller P Duran K Chen A Groot AJ Klomp LW. Increased activity of hypoxia-inducible factor 1 is associated with early embryonic lethality in Commd1 null mice. Mol Cell Biol (2007) 27:4142–56. 10.1128/MCB.01932-06 PMC1900009 17371845

[11] Burstein E Hoberg JE Wilkinson AS Rumble JM Csomos RA Komarck CM. COMMD proteins, a novel family of structural and functional homologs of MURR1. J Biol Chem (2005) 280:22222–32. 10.1074/jbc.M501928200 15799966

[12] Starokadomskyy P Gluck N Li H Chen B Wallis M Maine GN. CCDC22 deficiency in humans blunts activation of proinflammatory NF-kappaB signaling. J Clin Invest (2013) 123:2244–56. 10.1172/JCI66466 PMC3635737 23563313

[13] Biasio W Chang T McIntosh CJ McDonald FJ. Identification of Murr1 as a regulator of the human delta epithelial sodium channel. J Biol Chem (2004) 279:5429–34. 10.1074/jbc.M311155200 14645214

[14] Bartuzi P Billadeau DD Favier R Rong S Dekker D Fedoseienko A. CCC- and WASH-mediated endosomal sorting of LDLR is required for normal clearance of circulating LDL. Nat Commun (2016) 7:10961. 10.1038/ncomms10961 26965651 PMC4792963

[15] Campion CG Zaoui K Verissimo T Cossette S Matsuda H Solban N. COMMD5/HCaRG Hooks Endosomes on Cytoskeleton and Coordinates EGFR Trafficking. Cell Rep (2018) 24:670–84.e677. 10.1016/j.celrep.2018.06.056 30021164

[16] Li H Koo Y Mao X Sifuentes-Dominguez L Morris LL Jia D. Endosomal sorting of Notch receptors through COMMD9-dependent pathways modulates Notch signaling. J Cell Biol (2015) 211:605–17. 10.1083/jcb.201505108 PMC4639872 26553930

[17] Fedoseienko A Wieringa HW Wisman GB Duiker E Reyners AK Hofker MH. Nuclear COMMD1 Is Associated with Cisplatin Sensitivity in Ovarian Cancer. PLoS One (2016) 11:e0165385. 10.1371/journal.pone.0165385 27788210 PMC5082896

[18] Mu P Akashi T Lu F Kishida S Kadomatsu K. A novel nuclear complex of DRR1, F-actin and COMMD1 involved in NF-kappaB degradation and cell growth suppression in neuroblastoma. Oncogene (2017) 36:5745–56. 10.1038/onc.2017.181 28604741

[19] Zoubeidi A Ettinger S Beraldi E Hadaschik B Zardan A Klomp LW. Clusterin facilitates COMMD1 and I-kappaB degradation to enhance NF-kappaB activity in prostate cancer cells. Mol Cancer Res (2010) 8:119–30. 10.1158/1541-7786.MCR-09-0277 PMC2808437 20068069

[20] Yeh DW Chen YS Lai CY Liu YL Lu CH Lo JF. Downregulation of COMMD1 by miR-205 promotes a positive feedback loop for amplifying inflammatory- and stemness-associated properties of cancer cells. Cell Death Differ (2016) 23:841–52. 10.1038/cdd.2015.147 PMC4832103 26586569

[21] Fernandez Masso JR Oliva Arguelles B Tejeda Y Astrada S Garay H Reyes O. The Antitumor Peptide CIGB-552 Increases COMMD1 and Inhibits Growth of Human Lung Cancer Cells. J Amino Acids (2013) 2013:251398. 10.1155/2013/251398 23401744 PMC3562689

[22] Li H Burstein E. COMMD1 regulates inflammation and colitis-associated cancer progression. Oncoimmunology (2014) 3:e947891. 10.4161/21624011.2014.947891 25610735 PMC4292209

[23] Yang SS Li XM Yang M Ren XL Hu JL Zhu XH. FMNL2 destabilises COMMD10 to activate NF-kappaB pathway in invasion and metastasis of colorectal cancer. Br J Cancer (2017) 117:1164–75. 10.1038/bjc.2017.260 PMC5674093 28817833

[24] Zhan W Wang W Han T Xie C Zhang T Gan M. COMMD9 promotes TFDP1/E2F1 transcriptional activity *via* interaction with TFDP1 in non-small cell lung cancer. Cell Signal (2017) 30:59–66. 10.1016/j.cellsig.2016.11.016 27871936

[25] Dai H Ehrentraut S Nagel S Eberth S Pommerenke C Dirks WG. Genomic Landscape of Primary Mediastinal B-Cell Lymphoma Cell Lines. PLoS One (2015) 10:e0139663. 10.1371/journal.pone.0139663 26599546 PMC4657880

[26] Kwiecinska A Ichimura K Berglund M Dinets A Sulaiman L Collins VP. Amplification of 2p as a genomic marker for transformation in lymphoma. Genes Chromosomes Cancer (2014) 53:750–68. 10.1002/gcc.22184 PMC4369132 24832791

[27] Slovak ML Bedell V Hsu YH Estrine DB Nowak NJ Delioukina ML. Molecular karyotypes of Hodgkin and Reed-Sternberg cells at disease onset reveal distinct copy number alterations in chemosensitive versus refractory Hodgkin lymphoma. Clin Cancer Res (2011) 17:3443–54. 10.1158/1078-0432.CCR-10-1071 PMC3096736 21385932

[28] Taskinen M Louhimo R Koivula S Chen P Rantanen V Holte H. Deregulation of COMMD1 is associated with poor prognosis in diffuse large B-cell lymphoma. PLoS One (2014) 9:e91031. 10.1371/journal.pone.0091031 24625556 PMC3953211

[29] Kjeldsen E. Duplication of subtelomeric regions in an adult with acute monocytic leukemia with an acquired jumping translocation involving 3q13.31-qter. Data Brief (2017) 13:675–82. 10.1016/j.dib.2017.06.043 PMC5506863 28725672

[30] Mulaw MA Krause A Deshpande AJ Krause LF Rouhi A La Starza R. CALM/AF10-positive leukemias show upregulation of genes involved in chromatin assembly and DNA repair processes and of genes adjacent to the breakpoint at 10p12. Leukemia (2012) 26:1012–9. 10.1038/leu.2011.307 22064352

[31] Barresi V Trovato-Salinaro A Spampinato G Musso N Castorina S Rizzarelli E. Transcriptome analysis of copper homeostasis genes reveals coordinated upregulation of SLC31A1,SCO1, and COX11 in colorectal cancer. FEBS Open Bio (2016) 6:794–806. 10.1002/2211-5463.12060 PMC4971835 27516958

[32] Cornella H Alsinet C Sayols S Zhang Z Hao K Cabellos L. Unique genomic profile of fibrolamellar hepatocellular carcinoma. Gastroenterology (2015) 148:806–18.e810. 10.1053/j.gastro.2014.12.028 25557953 PMC4521774

[33] Peng CH Liao CT Peng SC Chen YJ Cheng AJ Juang JL. A novel molecular signature identified by systems genetics approach predicts prognosis in oral squamous cell carcinoma. PLoS One (2011) 6:e23452. 10.1371/journal.pone.0023452 21853135 PMC3154947

[34] Chen BL Yu J Zeng ZR Chu WK Wong CY Cheng YY. Rosiglitazone suppresses gastric carcinogenesis by up-regulating HCaRG expression. Oncol Rep (2008) 20:1093–7. 10.3892/or_00000114 18949406

[35] Devlin AM Solban N Tremblay S Gutkowska J Schurch W Orlov SN. HCaRG is a novel regulator of renal epithelial cell growth and differentiation causing G2M arrest. Am J Physiol Renal Physiol (2003) 284:F753–62. 10.1152/ajprenal.00252.2002 12620924

[36] El Hader C Tremblay S Solban N Gingras D Beliveau R Orlov SN. HCaRG increases renal cell migration by a TGF-alpha autocrine loop mechanism. Am J Physiol Renal Physiol (2005) 289:F1273–80. 10.1152/ajprenal.00103.2005 16033922

[37] Solban N Dumas P Gossard F Sun Y Pravenec M Kren V. Chromosomal mapping of HCaRG, a novel hypertension-related, calcium-regulated gene. Folia Biol (Praha) (2002) 48:9–14. 11871861 10.14712/fb2002048010009

[38] Solban N Jia HP Richard S Tremblay S Devlin AM Peng J. HCaRG, a novel calcium-regulated gene coding for a nuclear protein, is potentially involved in the regulation of cell proliferation. J Biol Chem (2000) 275:32234–43. 10.1074/jbc.M001352200 10918053

[39] Matsuda H Campion CG Fujiwara K Ikeda J Cossette S Verissimo T. HCaRG/COMMD5 inhibits ErbB receptor-driven renal cell carcinoma. Oncotarget (2017) 8:69559–76. 10.18632/oncotarget.18012 PMC5642500 29050225

[40] Matsuda H Hamet P Tremblay J. Hypertension-related, calcium-regulated gene (HCaRG/COMMD5) and kidney diseases: HCaRG accelerates tubular repair. J Nephrol (2014) 27:351–60. 10.1007/s40620-014-0054-3 PMC4104007 24515317

[41] Matsuda H Lavoie JL Gaboury L Hamet P Tremblay J. HCaRG accelerates tubular repair after ischemic kidney injury. J Am Soc Nephrol (2011) 22:2077–89. 10.1681/ASN.2010121265 PMC3279999 21921141

[42] Cerami E Gao J Dogrusoz U Gross BE Sumer SO Aksoy BA. The cBio cancer genomics portal: an open platform for exploring multidimensional cancer genomics data. Cancer Discov (2012) 2:401–4. 10.1158/2159-8290.CD-12-0095 PMC3956037 22588877

[43] Gao J Aksoy BA Dogrusoz U Dresdner G Gross B Sumer SO. Integrative analysis of complex cancer genomics and clinical profiles using the cBioPortal. Sci Signal (2013) 6:pl1. 10.1126/scisignal.2004088 23550210 PMC4160307

[44] Gudmundsson J Sulem P Gudbjartsson DF Masson G Petursdottir V Hardarson S. A common variant at 8q24.21 is associated with renal cell cancer. Nat Commun (2013) 4:2776. 10.1038/ncomms3776 24220699

[45] Ghoussaini M Song H Koessler T Al Olama AA Kote-Jarai Z Driver KE. Multiple loci with different cancer specificities within the 8q24 gene desert. J Natl Cancer Inst (2008) 100:962–6. 10.1093/jnci/djn190 PMC2902819 18577746

[46] Gudmundsson J Sulem P Manolescu A Amundadottir LT Gudbjartsson D Helgason A. Genome-wide association study identifies a second prostate cancer susceptibility variant at 8q24. Nat Genet (2007) 39:631–7. 10.1038/ng1999 17401366

[47] Yeager M Orr N Hayes RB Jacobs KB Kraft P Wacholder S. Genome-wide association study of prostate cancer identifies a second risk locus at 8q24. Nat Genet (2007) 39:645–9. 10.1038/ng2022 17401363

[48] Easton DF Pooley KA Dunning AM Pharoah PD Thompson D Ballinger DG. Genome-wide association study identifies novel breast cancer susceptibility loci. Nature (2007) 447:1087–93. 10.1038/nature05887 PMC2714974 17529967

[49] Pei YL Zhang HL Han HG. Polymorphism of 8q24 rsl3281615 and breast cancer risk: a meta-analysis. Tumour Biol (2013) 34:421–8. 10.1007/s13277-012-0566-1 23132293

[50] Turnbull C Ahmed S Morrison J Pernet D Renwick A Maranian M. Genome-wide association study identifies five new breast cancer susceptibility loci. Nat Genet (2010) 42:504–7. 10.1038/ng.586 PMC3632836 20453838

[51] Kang JU. Chromosome 8q as the most frequent target for amplification in early gastric carcinoma. Oncol Lett (2014) 7:1139–43. 10.3892/ol.2014.1849 PMC3961457 24944681

[52] Ahmadiyeh N Pomerantz MM Grisanzio C Herman P Jia L Almendro V. 8q24 prostate, breast, and colon cancer risk loci show tissue-specific long-range interaction with MYC. Proc Natl Acad Sci U S A (2010) 107:9742–6. 10.1073/pnas.0910668107 PMC2906844 20453196

[53] Hutter CM Slattery ML Duggan DJ Muehling J Curtin K Hsu L. Characterization of the association between 8q24 and colon cancer: gene-environment exploration and meta-analysis. BMC Cancer (2010) 10:670. 10.1186/1471-2407-10-670 21129217 PMC3017062

[54] Zanke BW Greenwood CM Rangrej J Kustra R Tenesa A Farrington SM. Genome-wide association scan identifies a colorectal cancer susceptibility locus on chromosome 8q24. Nat Genet (2007) 39:989–94. 10.1038/ng2089 17618283

[55] Han J Zhou J Yuan H Zhu L Ma H Hang D. Genetic variants within the cancer susceptibility region 8q24 and ovarian cancer risk in Han Chinese women. Oncotarget (2017) 8:36462–8. 10.18632/oncotarget.16861 PMC5482668 28430593

[56] White KL Sellers TA Fridley BL Vierkant RA Phelan CM Tsai YY. Variation at 8q24 and 9p24 and risk of epithelial ovarian cancer. Twin Res Hum Genet (2010) 13:43–56. 10.1375/twin.13.1.43 20158306 PMC2932441

[57] Kiemeney LA Thorlacius S Sulem P Geller F Aben KK Stacey SN. Sequence variant on 8q24 confers susceptibility to urinary bladder cancer. Nat Genet (2008) 40:1307–12. 10.1038/ng.229 PMC4539560 18794855

[58] Crowther-Swanepoel D Broderick P Di Bernardo MC Dobbins SE Torres M Mansouri M. Common variants at 2q37.3, 8q24.21, 15q21.3 and 16q24.1 influence chronic lymphocytic leukemia risk. Nat Genet (2010) 42:132–6. 10.1038/ng.510 PMC5321238 20062064

[59] Beroukhim R Mermel CH Porter D Wei G Raychaudhuri S Donovan J. The landscape of somatic copy-number alteration across human cancers. Nature (2010) 463:899–905. 10.1038/nature08822 20164920 PMC2826709

[60] Moore SR Persons DL Sosman JA Bobadilla D Bedell V Smith DD. Detection of copy number alterations in metastatic melanoma by a DNA fluorescence *in situ* hybridization probe panel and array comparative genomic hybridization: a southwest oncology group study (S9431). Clin Cancer Res (2008) 14:2927–35. 10.1158/1078-0432.CCR-07-4068 18483359

[61] Nowak NJ Miecznikowski J Moore SR Gaile D Bobadilla D Smith DD. Challenges in array comparative genomic hybridization for the analysis of cancer samples. Genet Med (2007) 9:585–95. 10.1097/GIM.0b013e3181461c4a 17873646

[62] Hirashima K Migita T Sato S Muramatsu Y Ishikawa Y Seimiya H. Telomere length influences cancer cell differentiation *in vivo*. Mol Cell Biol (2013) 33:2988–95. 10.1128/MCB.00136-13 PMC3719673 23716593

[63] Herranz N Gil J. Mechanisms and functions of cellular senescence. J Clin Invest (2018) 128:1238–46. 10.1172/JCI95148 PMC5873888 29608137

[64] Campisi J. Cellular Senescence, Aging and Cancer. Sci World J (2001) 1:65. 10.1100/tsw.2001.106 PMC6084413 30147535

[65] Pucci F Gardano L Harrington L. Short telomeres in ESCs lead to unstable differentiation. Cell Stem Cell (2013) 12:479–86. 10.1016/j.stem.2013.01.018 PMC3629568 23561444

[66] Westhoff JH Schildhorn C Jacobi C Homme M Hartner A Braun H. Telomere shortening reduces regenerative capacity after acute kidney injury. J Am Soc Nephrol (2010) 21:327–36. 10.1681/ASN.2009010072 PMC2834551 19959722

[67] Holzmann K Blin N Welter C Zang KD Seitz G Henn W. Telomeric associations and loss of telomeric DNA repeats in renal tumors. Genes Chromosomes Cancer (1993) 6:178–81. 10.1002/gcc.2870060308 7682103

[68] Mehle C Ljungberg B Roos G. Telomere shortening in renal cell carcinoma. Cancer Res (1994) 54:236–41. 8261445

[69] Fiedler W Dahse R Schlichter A Junker K Kosmehl H Ernst G. Telomerase activity and telomere length in different areas of renal cell carcinoma. Int J Oncol (1996) 9:1227–32. 10.3892/ijo.9.6.1227 21541632

[70] Dahse R Fiedler W Junker K Schlichter A Schubert J Claussen U. Telomerase activity and telomere lengths: alterations in renal cell carcinomas. Kidney Int (1999) 56:1289–90. 10.1046/j.1523-1755.1999.00688.x 10504477

[71] Morais M Dias F Teixeira AL Medeiros R. Telomere Length in Renal Cell Carcinoma: The Jekyll and Hyde Biomarker of Ageing of the Kidney. Cancer Manag Res (2020) 12:1669–79. 10.2147/CMAR.S211225 PMC7064280 32184670

[72] Svenson U Ljungberg B Roos G. Telomere length in peripheral blood predicts survival in clear cell renal cell carcinoma. Cancer Res (2009) 69:2896–901. 10.1158/0008-5472.CAN-08-3513 19318563

[73] Pal D Sharma U Khajuria R Singh SK Kakkar N Prasad R. Augmented telomerase activity, reduced telomere length and the presence of alternative lengthening of telomere in renal cell carcinoma: plausible predictive and diagnostic markers. Gene (2015) 562:145–51. 10.1016/j.gene.2015.02.079 25769384

[74] Wills LP Schnellmann RG. Telomeres and telomerase in renal health. J Am Soc Nephrol (2011) 22:39–41. 10.1681/ASN.2010060662 21209253

[75] McNally EJ Luncsford PJ Armanios M. Long telomeres and cancer risk: the price of cellular immortality. J Clin Invest (2019) 129:3474–81. 10.1172/JCI120851 PMC6715353 31380804

[76] Kim NW Piatyszek MA Prowse KR Harley CB West MD Ho PL. Specific association of human telomerase activity with immortal cells and cancer. Science (1994) 266:2011–5. 10.1126/science.7605428 7605428

[77] Recagni M Bidzinska J Zaffaroni N Folini M. The Role of Alternative Lengthening of Telomeres Mechanism in Cancer: Translational and Therapeutic Implications. Cancers (Basel) (2020) 12(4):949. 10.3390/cancers12040949 PMC7226354 32290440

[78] Rampazzo E Bertorelle R Serra L Terrin L Candiotto C Pucciarelli S. Relationship between telomere shortening, genetic instability, and site of tumour origin in colorectal cancers. Br J Cancer (2010) 102:1300–5. 10.1038/sj.bjc.6605644 PMC2856015 20386541

[79] Robin JD Ludlow AT Batten K Magdinier F Stadler G Wagner KR. Telomere position effect: regulation of gene expression with progressive telomere shortening over long distances. Genes Dev (2014) 28:2464–76. 10.1101/gad.251041.114 PMC4233240 25403178

[80] Robin JD Ludlow AT Batten K Gaillard MC Stadler G Magdinier F. SORBS2 transcription is activated by telomere position effect-over long distance upon telomere shortening in muscle cells from patients with facioscapulohumeral dystrophy. Genome Res (2015) 25:1781–90. 10.1101/gr.190660.115 PMC4665000 26359233

[81] Kim W Shay JW. Long-range telomere regulation of gene expression: Telomere looping and telomere position effect over long distances (TPE-OLD). Differentiation (2018) 99:1–9. 10.1016/j.diff.2017.11.005 29197683 PMC5826875

[82] Kim W Ludlow AT Min J Robin JD Stadler G Mender I. Regulation of the Human Telomerase Gene TERT by Telomere Position Effect-Over Long Distances (TPE-OLD): Implications for Aging and Cancer. PLoS Biol (2016) 14:e2000016. 10.1371/journal.pbio.2000016 27977688 PMC5169358

[83] Lou Z Wei J Riethman H Baur JA Voglauer R Shay JW. Telomere length regulates ISG15 expression in human cells. Aging (Albany NY) (2009) 1:608–21. 10.18632/aging.100066 PMC2806043 20157543

[84] Pedram M Sprung CN Gao Q Lo AW Reynolds GE Murnane JP. Telomere position effect and silencing of transgenes near telomeres in the mouse. Mol Cell Biol (2006) 26:1865–78. 10.1128/MCB.26.5.1865-1878.2006 PMC1430234 16479005

[85] Zhou BO Wang SS Zhang Y Fu XH Dang W Lenzmeier BA. Histone H4 lysine 12 acetylation regulates telomeric heterochromatin plasticity in Saccharomyces cerevisiae. PLoS Genet (2011) 7:e1001272. 10.1371/journal.pgen.1001272 21249184 PMC3020936

[86] Weuts A Voet T Verbeeck J Lambrechts N Wirix E Schoonjans L. Telomere length homeostasis and telomere position effect on a linear human artificial chromosome are dictated by the genetic background. Nucleic Acids Res (2012) 40:11477–89. 10.1093/nar/gks926 PMC3526267 23066103

[87] Buxton JL Suderman M Pappas JJ Borghol N McArdle W Blakemore AI. Human leukocyte telomere length is associated with DNA methylation levels in multiple subtelomeric and imprinted loci. Sci Rep (2014) 4:4954. 10.1038/srep04954 24828261 PMC4344300

[88] Tennen RI Bua DJ Wright WE Chua KF. SIRT6 is required for maintenance of telomere position effect in human cells. Nat Commun (2011) 2:433. 10.1038/ncomms1443 21847107 PMC3528101

[89] Anderson MZ Gerstein AC Wigen L Baller JA Berman J. Silencing is noisy: population and cell level noise in telomere-adjacent genes is dependent on telomere position and sir2. PLoS Genet (2014) 10:e1004436. 10.1371/journal.pgen.1004436 25057900 PMC4109849

[90] Campisi J. Cancer, aging and cellular senescence. In Vivo (2000) 14:183–8. 10.1146/annurev-physiol-030212-183653 10757076

[91] Cristofalo VJ Pignolo RJ. Replicative senescence of human fibroblast-like cells in culture. Physiol Rev (1993) 73:617–38. 10.1152/physrev.1993.73.3.617 8332640

[92] Cristofalo VJ Pignolo RJ. Molecular markers of senescence in fibroblast-like cultures. Exp Gerontol (1996) 31:111–23. 10.1016/0531-5565(95)02018-7 8706781

[93] Hayflick L. The Limited in Vitro Lifetime of Human Diploid Cell Strains. Exp Cell Res (1965) 37:614–36. 10.1016/0014-4827(65)90211-9 14315085

[94] Xu D Takeshita F Hino Y Fukunaga S Kudo Y Tamaki A. miR-22 represses cancer progression by inducing cellular senescence. J Cell Biol (2011) 193:409–24. 10.1083/jcb.201010100 PMC3080260 21502362

